# Three-Dimensional Epitaxy of Low-Defect 3C-SiC on a Geometrically Modified Silicon Substrate

**DOI:** 10.3390/ma17071587

**Published:** 2024-03-30

**Authors:** Gerard Colston, Kelly Turner, Arne Renz, Kushani Perera, Peter M. Gammon, Marina Antoniou, Vishal A. Shah

**Affiliations:** School of Engineering, The University of Warwick, Coventry CV4 7AL, UK; g.colston.1@warwick.ac.uk (G.C.); kelly.turner@warwick.ac.uk (K.T.); arne.renz@warwick.ac.uk (A.R.); kushani-hapuhinna.perera@warwick.ac.uk (K.P.); p.m.gammon@warwick.ac.uk (P.M.G.); marina.antoniou@warwick.ac.uk (M.A.)

**Keywords:** 3C-SiC, epitaxy, compliant substrate, stacking faults, conformal epitaxy

## Abstract

We demonstrate the growth of 3C-SiC with reduced planar defects on a micro-scale compliant substrate. Heteroepitaxial growth of 3C-SiC on trenches with a width and separation of 2 µm, etched into a Si(001) substrate, is found to suppress defect propagation through the epilayer. Stacking faults and other planar defects are channeled away from the center of the patterned structures, which are rounded through the use of H_2_ annealing at 1100 °C. Void formation between the columns of 3C-SiC growth acts as a termination point for defects, and coalescence of these columns into a continuous epilayer is promoted through the addition of HCl in the growth phase. The process of fabricating these compliant substrates utilizes standard processing techniques found within the semiconductor industry and is independent of the substrate orientation and offcut.

## 1. Introduction

Silicon carbide (SiC) is a wide-bandgap compound semiconductor with high electrical field breakdown and thermal conductivity, which makes it ideal for high-voltage power electronics and other applications in harsh environments [1]. SiC can exist in a number of different crystalline forms due to the stacking sequence of the C-Si bilayers; these are known as polytypes. The hexagonal structured 4H-SiC is highly mature and is available as single crystal substrates, which when paired with high-temperature homoepitaxial growth, form the basis for commercially available power electronic devices. The cost of this material, however, both in terms of the starting substrate and epitaxy, is extremely high and limits its uptake in mass markets [2].

The cubic form of SiC (3C-SiC) has a zinc-blende (diamond) structure and stabilizes at lower temperatures than other polytypes, allowing it to be grown on silicon (Si). The heteroepitaxial growth of 3C-SiC offers many advantages such as low-cost, large-diameter and high-quality starting Si substrates as well as low epitaxial growth temperatures [3,4]. 3C-SiC is of particular importance to power electronics in the 600–1200 V range, as it has a low built-in voltage [5], which accounts for the low specific on-resistance (R_on_._sp_) losses and large 3C-SiC/SiO_2_ barrier, which potentially support devices’ reliability [6].

However, the heteroepitaxial growth of 3C-SiC on Si is plagued by two main difficulties. First, the large lattice mismatch (19.7%) between 3C-SiC and Si introduces a high density of misfit dislocations at the interface and stacking faults that propagate up throughout the epilayer [7]. Stacking faults and other planar defects including micro-twins and anti-phase domains are electrically active and cause high levels of leakage currents in rectifying devices [8,9]. The second issue with heteroepitaxial growth is the high thermal mismatch (8–20%) between 3C-SiC and the Si substrate, which can lead to high levels of thermal stress and wafer bow, which increase with epilayer thickness and can leave epi wafers unsuitable for device fabrication processes [10].

Defect densities can be reduced in 3C-SiC through the epitaxial growth of thicker films, due to the self-annihilation of planar defects; however, this increases epitaxy costs and often increases wafer bow. In addition, this method only reduces the defect density of the 3C-SiC at the surface and does not improve the crystallinity of the epilayer near the interface with the substrate.

More novel processes have been explored to reduce the defectiveness of 3C-SiC, such as the production of substrates suitable for homoepitaxy [11]. Other options include modifying the structure of the starting Si substrate to suppress defect formation and promote the annihilation process. Additionally, there are techniques such as patterning the growth on undulant silicon using diamond slurry [12], growing 3C-SiC on fine-structured nm-scale hexagonal pillars [13] and trapping defects within inverted Si pyramids [14]. The growth of 3C-SiC on porous Si has also been explored to improve adhesion and reduce lattice mismatch between the thin film and substrate [15]. The use of compliant substrates such as these offers a reduction in defects and also the potential to suppress wafer bow; however, several of these methods rely on precise fabrication techniques that are dependent on the substrate’s crystal orientation. Any growth on isolated structures also suffers from issues with coalescence of the islands into a continuous film and the introduction of further defects such as anti-phase boundaries [13].

The surface profile of the structures on compliant substrates has been found to have an important impact on the control of defect propagation, as evidenced by growth on pyramids or an undulant surface. Flat surfaces and vertical sidewalls may not offer the ideal template for 3C-SiC growth; however, even with extremely fine patterning processes such as electron-beam lithography, it is often impossible to eliminate the flat regions of the substrate in compliant substrates. Additional processing steps may be necessary to further modify the structure of the patterned silicon prior to epitaxy. One method for achieving this is through annealing processes. The thermal annealing of Si structures in a H_2_ atmosphere causes the surface diffusion of Si atoms and can be used to smooth and round Si patterns. The extent of this rounding can be controlled with both the annealing temperature and time [16]. This study investigates the impact that such surface modification has on the heteroepitaxial growth of 3C-SiC on a patterned Si substrate.

## 2. Materials and Methods

Trenches of 2 µm width and 2 µm separation (defined as 4 µm pitch) were etched to a depth of approximately 4 µm into on-axis 100 mm diameter Si(001) substrates using UV photolithography and reactive ion etch (RIE) processing, with an SF_6_ and O_2_ plasma. The trenches were aligned to the <110> crystal plane directions. Photoresist was removed by solvent cleaning in acetone before being subjected to an RCA clean. The wafers were then loaded into an epitaxial reactor, ensuring no contamination was introduced into the growth system, and the substrate surface was primed for epitaxy.

Epitaxial growth and thermal annealing were performed within an LPE ACIS-M8 reduced-pressure chemical vapor deposition (RP-CVD) reactor at the University of Warwick. 3C-SiC films were grown at a temperature of 1325 °C using trichlorosilane (TCS, SiHCl_3_) and ethylene (C_2_H_4_) with a C/Si ratio of 1.4, within a H_2_ carrier gas at a growth rate of ~6 µm/h. A carbonization process was used to initiate the growth of the 3C-SiC, which involved the deposition of a thin seed layer using C_2_H_4_ at a temperature range starting at 900 °C and ramping up to 1140 °C. On selected samples, the surface profile of the trench walls was modified using H_2_ annealing at 1100 °C for 10 min prior to a carbonization process.

The grown 3C-SiC epilayers were characterized by scanning electron microscopy (SEM) and cross-sectional transmission electron microscopy (TEM), with transparent electron cross-sections extracted and polished using a focused ion bean scanning electron microscope (FIB-SEM). The FIB-SEM process was necessary to extract cross-sections from specific locations on the epi wafers.

## 3. Results

### 3.1. Annealing of Si Trenches

**Figure 1:**
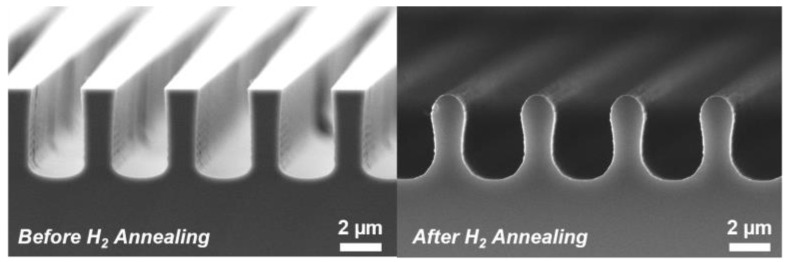
Cross-sectional SEM micrographs highlighting the rounding of the Si trench profile before and after being subject to 1100 °C H_2_ annealing.

Dry etching of silicon can lead to unwanted features such as striations along trench walls and micro-trenches in the base of a trench due to limitations in UV photolithography and imperfect dry etching processes [17]. Annealing the trenches in H_2_ modifies the imperfect profile of the mesa walls, giving them a smooth surface, and thereby eliminates these features. Annealing at a sufficiently high temperature can facet and round the surfaces of the Si mesas. Samples not exposed to this baking retain their flat (001)-orientation surface and base and {110} sidewalls (see Figure 1). In this study, the H_2_ annealing process was found to have a minimal effect on the trench depth, with the sample depths measured at 4.2 µm and 4.1 µm before and after annealing, respectively.

### 3.2. 3C-SiC Epitaxy

**Figure 2:**
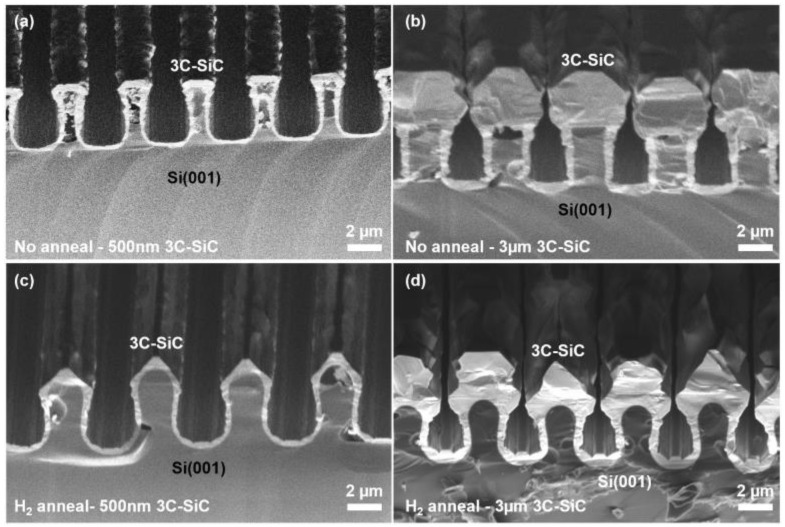
Cross-sectional SEM micrographs of 500 nm and 3 µm 3C-SiC heteroepitaxially grown on Si trench structures without annealing (**a**,**b**) and after 1100 °C H_2_ annealing (**c**,**d**).

Conformal epitaxial growth of thin layers of 3C-SiC was found in the Si trenches and on the sidewalls for thin epilayers; however, as the epilayer thickness increased, the sidewall growth was suppressed and voids formed within and above the trenches (see Figure 2). We speculate that this was due to the shadowing effect of the epilayers growing on top of the structures, preventing the flow of precursors into the underlying trenches. Growth on the Si structures that were rounded through thermal annealing appeared more disordered than on the unannealed structures and, in either case, no fusion of the separated epilayers was observed at a growth thickness of 3 µm.

**Figure 3:**
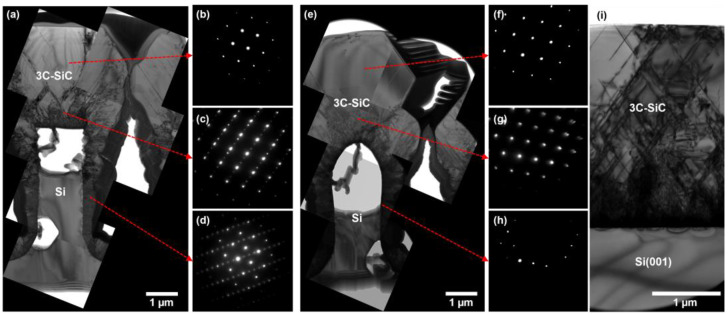
Cross-sectional TEM micrographs of 3 µm 3C-SiC grown on Si trench sidewalls without (**a**) and with 1100 °C H_2_ annealing (**e**). SAED patterns of 3C-SiC grown on both structures confirm that the material above the sidewall profile is monocrystalline (**b**,**f**) while 3C-SiC on the surfaces and sidewalls of the structures shows elements of polycrystallinity and defectiveness (**c**,**d**,**g**,**h**). 3C-SiC grown directly on a Si(001) substrate is shown in (**i**) for comparison.

Cross-sectional TEM showed that the 3C-SiC grown on both rounded and non-rounded structures was crystalline; however, a clear difference in defect density could be observed between the samples (see Figure 3). The rounded surface of the annealed structure was observed to direct stacking faults away from the center of the peak, resulting in an almost defect-free region above the Si structure (see Figure 3e). The selective area electron diffraction (SAED) patterns showed that while the material close to the Si structure had elements of hexagonality caused by the high density of stacking faults within the epilayer, the 3C-SiC growing above the Si structure was monocrystalline in both cases. A clear interface could be observed between adjacent structures, showing that the layers had not coalesced, and, in both cases, the sides of the 3C-SiC growth columns were highly disordered.

**Figure 4:**
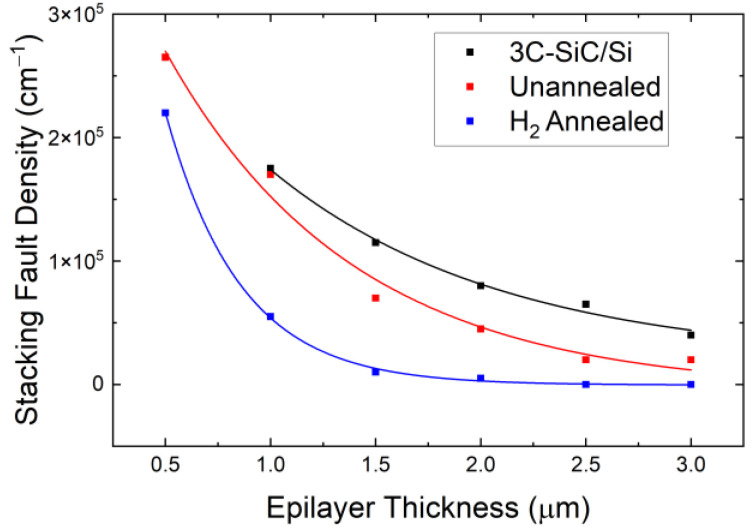
Linear stacking fault density calculated from TEM micrographs at varying depths. The density of defects for patterned wafers is taken directly above the Si structures.

The stacking fault density in the region above the pillars of each sample was estimated from TEM images and compared to that of the growth on non-patterned Si (see Figure 4). The density of stacking faults, based on these local measurements, was found to decrease for the patterned structures, with a further significant reduction after H_2_ annealing through the channeling of planar defects away from the center of the mesa.

### 3.3. Improving Coalescence with HCl

**Figure 5:**
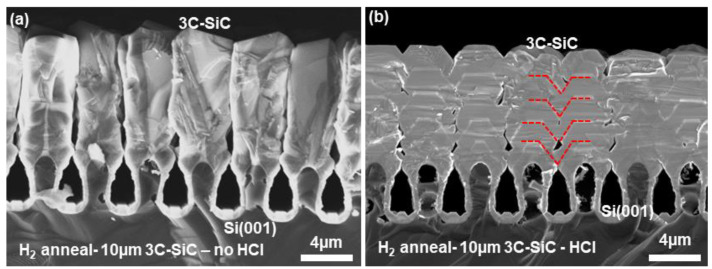
Cross-sectional SEM micrograph of 10 µm 3C-SiC grown on Si trench structures subject to 1100 °C H_2_ annealing without HCl (**a**) and with additional HCl (**b**) during epitaxy. The growth fronts of one column are highlighted in red to show the growth evolution.

One key limitation of the method described so far is the inability of individual 3C-SiC columns to fuse with their neighbors and create a coherent epilayer. It has been proposed that the lack of coalescence of these 3C-SiC films may be due to their thinness; however, increasing the growth of 3C-SiC to 10 µm did not trigger any fusion. Instead, separate individual columns of 3C-SiC grew upon each of the Si structures (see Figure 5a). In another idea, the disordered structure of 3C-SiC is thought to cause the lack of coalescence, and so 500 sccm of HCl was introduced into the growth process of 3C-SiC to assess the impact of this etching agent. At typical growth temperatures of 3C-SiC, HCl is found to etch highly dislocated regions of material faster than ordered crystal areas and, hence, can be used to promote the faceted growth of 3C-SiC [18]. In the case of growth on Si(001), the addition of sufficient HCl increased the prevalence of the 3C(001) and 3C(111) surfaces. When these regions met, they were observed to coalesce much more effectively (see Figure 5b). Al doping markers were grown in situ during the epitaxial growth of the 10 µm 3C-SiC epilayer with additional HCl at every 2 µm of growth to highlight the growth evolution of the film when observed by SEM. Even with the H_2_ annealing process, the growth fronts were found to be the (001) and (111) crystal planes, and the length of (001) was found to increase as the epilayer increased in thickness, indicating the fusion of the (111) planes. Were this epilayer to be grown up to >10 µm, a flat epilayer surface may be achieved, as the (111) planes may fuse together entirely.

Further optimization of this growth process is required to improve the coalescence of these columns and to improve the surface morphology of the layer. Increasing the flow of HCl will impact the prevalence on different 3C-SiC growth fronts; however, improvement could also be achieved by modifying the aspect ratio of the mesas and trench separation. Chemical mechanical polishing (CMP) could be employed to give a smooth top surface, making the material suitable for further epitaxy, wafer bonding or device fabrication, as well as producing a high density of voids at the 3C-SiC/Si interfaces on the patterned structures due to the increased Si surface area.

## 4. Discussion

The growth of 3C-SiC on patterned substrates leads to a reduction in the stacking fault density by trapping defects at the sidewalls of 3C-SiC columns. A further reduction in defect density is obtained by modifying the geometry of the Si structures, which can be achieved through thermal annealing in H_2_. Rounding the starting Si structure is shown to generate planar defects, which propagate along the {111} crystal planes that direct away from the [001] growth direction. Conformal epitaxial growth of 3C-SiC on such micro-structures begins to form on Si; however, once a thickness comparable to the separation between structures is obtained, growth within the trenches is suppressed and voids are formed. These voids act as natural defect termination points and may help reduce thermally induced wafer bow by breaking up the continuous interface of 3C-SiC/Si.

The challenge with the growth of epilayers on separate structures such as this is promoting the coalescence of the individual columns into a continuous film; however, this has been achieved through the introduction of sufficiently high levels of HCl into RP-CVD during epitaxy, which results in more ordered growth and enables the fusion of layers grown on the Si structures. A similar technique is used in the process of 4H-SiC trench refill epitaxy, whereby additional HCl is added to the epitaxial process to modify the growth and etch rates of 4H-SiC on different faces of the patterned structure [19,20].

Using a patterned substrate for the epitaxial growth of 3C-SiC has a clear impact on the formation of planar defects such as stacking faults and micro-twins; however, its impact on the generation of point defects is unknown. The formation of point defects in SiC is mainly controlled by the growth rate and temperature as well as the precursor composition and, as such, compliant substrates have little impact on point defect generation directly [14]; however, the change in stacking sequence caused by planar defects can lead to the formation of interstitials, which may be reduced by this technique [21].

## 5. Conclusions

The density of planar defects in 3C-SiC can be reduced through the use of the demonstrated micron-scale compliant substrate fabricated by the formation of trenches in a Si(001) substrate followed by thermal annealing in a H_2_ atmosphere. The rounded shape of the Si structure, formed by this annealing process, assists in channeling stacking faults away from the center of each pillar, thus resulting in an area of lower defect density. Such areas of 3C-SiC must then fuse with adjacent growth columns to produce a coalesced thin film, which can be enabled through the selective etching of additional HCl added into the growth phase. The pattern used in this study included linear trenches, which limited the investigation to the propagation of defects in one dimension; however, the technique could be expanded to arrays of pillars, which would enable defect trapping in both in-plane directions. The method presented offers a low-cost and highly scalable process for the fabrication of micron-scale compliant substrates for 3C-SiC as it relies solely on standard semiconductor processing techniques including photolithography and dry etching. The process does not rely on the crystal orientation and hence could be applied to other substrates beyond the (001) orientation and with various levels of offcut, which would help suppress the formation of anti-phase domains.

## Data Availability

The research data will be made available upon a reasonable request to the corresponding author.
